# Self-body recognition and attitudes towards body image in younger and older women

**DOI:** 10.1007/s00737-021-01164-x

**Published:** 2021-07-31

**Authors:** Ashleigh Bellard, Cosimo Urgesi, Valentina Cazzato

**Affiliations:** 1 School of Psychology, Faculty of Health, Liverpool John Moores University, Liverpool, UK; 2 Laboratory of Cognitive Neuroscience, Department of Language and Literature, Communication, Education and Society, University of Udine, Udine, Italy; 3 Scientific Institute, IRCCS E. Medea, Pasian di Prato, Udine, Italy

**Keywords:** Self-body knowledge, Implicit processing, Body uneasiness, Body parts concerns, Ageing

## Abstract

Despite the fact that ageing causes dramatic changes in bodily appearance, little is known about how self-body recognition changes across life span. Here, we investigated whether older, compared to younger women, differed in the ability of recognising their own than other women’s body parts and whether this effect was associated to negative body image dispositions. Twenty-eight young (M_age_: 25.93 years, SD_age_ = 4.74) and 25 middle-aged (M_age_: 54.36 years, SD_age_ = 4.54) women completed an implicit task consisting of visual matching of self and others’ body parts and an explicit self–other body discrimination task. Stimuli comprised of images of body parts of the participant and of other age- and BMI-matched models, which were presented in the original size or modified to look rounder or thinner. Measures of adiposity (i.e. BMI), body image concerns and appearance-related worries for specific body parts and for the whole body were also collected. Whilst both groups showed a self-body advantage in the implicit, but not in the explicit task, the advantage was notably bigger for the younger group. However, the implicit self-advantage was higher in those middle-aged women that displayed more body image concerns and worries for specific body parts. Furthermore, the two groups were comparably less able in recognising their body parts when presented thinner as compared to rounder or in their actual size. Overall, these findings open the possibility that, as women age, their implicit self-recognition abilities may decline in association with more negative body image dispositions.

**Supplementary Information:**

The online version contains supplementary material available at 10.1007/s00737-021-01164-x.

## Introduction

Self-body recognition, which refers to the unique ability in identifying one’s own body and its parts as separate from others (Richetin et al. 2012), allows for self–other discrimination, a pivotal cognitive function in social interaction and bodily self-awareness (Conson et al. 2010; Uddin et al. 2006). Self-body perception is based on separated (or partially overlapping) cognitive processes and neural systems, as compared to those used for processing others’ bodies. Indeed, we come to understand our body as a separate entity from someone else’s body, thanks to the complex operation by which we integrate multiple sources of information coming from inside (e.g. interoception) and outside (e.g. exteroception) of our body (Tsakiris 2017). As suggested by Myers and Sowden (2008), self-body knowledge, indeed, relies upon the integration of multiple sensory modalities including visceral, visual, somatosensory, proprioceptive, and motor information, to guide our interpretation of sensory events and our actions upon the world. Conversely, to distinguish other individual’s body parts, we may rely solely on visual information.

Former behavioural investigations have offered insight into the so-called self-body advantage, in which people provide faster (and more accurate) responses to self-body parts as opposed to others’ body parts (Frassinetti et al. 2011). This advantage does not only occur for visual body processing, but it also extends to other modalities, like self-voice recognition (Candini et al. 2014). In a series of studies, Frassinetti and colleagues (Frassinetti et al. 2008, 2009, 2010) showed that this superior ability in recognising the self emerges when the task requires an implicit access to self bodily knowledge (i.e. self-recognition is task-irrelevant), but not when an explicit self–other discrimination is required. For example, in a study conducted by Frassinetti et al. (2008), participants were presented with a two-alternative forced choice (2AFC) visual matching task, in which they were asked to report which of two body part images presented at the top or at the bottom of the screen matched a central body part stimulus. The matching and non-matching stimuli depicted the same specific body part, namely hand, foot, arm or leg, but belonging to different persons, either the participant or other gender- and age-matched models. Results demonstrated that participants were more accurate in matching when either the matching or non-matching body part belonged to them, as opposed to another person, thus revealing an implicit self-body processing advantage. In contrast, a self-body advantage was not found when an explicit recognition of one’s own vs others’ body parts was required (Frassinetti et al. 2011). Whilst patients with right hemisphere lesions, as compared to healthy controls and patients with left hemisphere lesions, were impaired in both the implicit and the explicit self-body processing tasks, the deficits in the two tasks were associated with damage to different regions of the right hemisphere (Frassinetti et al. 2008). Taken all together, these studies support the notion of a two-way access to self-body knowledge, which involves separate cognitive processes and neural mechanisms. Indeed, whilst a sensorimotor body representation (including proprioceptive and motor information) is engaged in the implicit recognition of one’s own body parts, the explicit recognition of one’s own body parts might be instead merely supported by a visual perceptual facilitation (Ferri et al. 2012).

### Ageing and self-body recognition

Age-related changes in the physical aspect of the body (for, e.g. appearance of wrinkles, grey hair or weight gain), accompanied by sensorimotor deterioration (Bullock-Saxton et al. 2001) and the individual dispositions towards these changes, may deeply affect self-body recognition and the sense of the body as being mine (body ownership). Compared to younger, older women and men undergo physical body changes which are naturally associated with ageing, including increased body mass index (BMI), weight gain (in body weight/fat distribution) and decrease in muscle mass. This in turn might lead to greater levels of body dissatisfaction, which also seem to remain quite stable across the adult lifespan in both women (Tiggemann and McCourt 2013; Tiggemann 2004) and men (Quittkat et al. 2019). In contrast, other studies suggest that older women do not always experience body preoccupations (Tiggemann and McCourt 2013) and instead show a more positive body image (Tylka 2011) than younger women, which could be due to the fact that, with increasing age, women shift their focus to and become more appreciative of their health and functionality rather than their physical appearance (Augustus-Horvath and Tylka 2011; Tiggemann and Lynch 2001). For men, several studies suggest that age might moderate the relationship between gender role conflict and muscle and body-fat dissatisfaction (Murray and Lewis 2014; Quittkat et al. 2019). Other investigations instead suggest that they are less likely to spend more hours per day on their ideal appearance than women and that, with higher age, men report lower levels of body appreciation compared to younger men and women (Quittkat et al. 2019). Nevertheless, in this logic, self-body recognition heavily depends on the ‘updated’ representation of one’s own physical appearance and functioning, which in turn might affect the ability to regard one’s own corporeal identity as separate from that of other individuals.

To date, the literature on how changes in physical appearance associated with ageing might affect self-body recognition has only focused on the sense of ‘body ownership’, which is thought to arise from the integration of current multisensory information (e.g. touch and vision) in internal models of the body and is mainly assessed by means of the Rubber-Hand Illusion. In this illusion, the vision of a rubber hand being touched in synchrony with the tactile stimulation of the real hand captures the feeling of body ownership, leading the participant to feel the seen touch (for review see Apps and Tsakiris 2013; Blanke 2012; Tsakiris 2010). A study by Marotta et al. (2018) reported that the mechanisms underlying the illusory embodiment of the rubber hand may change across the life span. Middle-aged adults were found to be more resistant to the body ownership illusion, suggesting less visual capture of body ownership and lower malleability of body representation compared to both younger and older adults (see also Graham et al. 2015 and Kállai et al. 2017 for converging evidence, but Palomo et al. 2018 for contrasting findings). No study, however, has so far tested whether ageing changes a purely visual representation of the bodily self in the absence of any concurrent somatic information. Indeed, to successfully recognise their own body parts in a picture, observers must compare the displayed picture with the mental representation of their own body, using visual cues and the information arising from memory. If this representation is affected by perceptual distortions, such as overestimation of body size or altered body shape perception, it might be the case that also self-body recognition is in turn affected. Furthermore, previous studies have solely limited to the recognition and embodiment of one body part (Frassinetti et al. 2010), namely the hand (Candini et al. 2016), which notably is not as salient in the evaluation of body appearance as other body parts, such as the abdomen, which instead is usually linked to worry of fatness and dissatisfaction in both the general population and in individuals experiencing disordered eating (Keizer et al. 2011, 2012, 2016; Spitoni et al. 2015; Bellard et al. 2020; Ralph-Nearman et al. 2019, 2021).

### The current study

To address these issues, we presented two groups of younger and older women with actual or size-distorted pictures of their own or other women’s body parts. We used grey-scaled images of varying levels of fatness (3 levels: round, slim and actual) of several body parts (i.e. stomach, foot, hand, thighs). All identifiable marks, i.e. birthmarks, moles, tattoos, scars, piercings and nail painting, were removed to avoid participants using these to identify their body parts. Self-body processing in both groups was probed using either an implicit or an explicit 2AFC self-processing task. For the implicit (covert) self-processing, in a delayed matching-to-sample task, participants were presented with a sequence of two body parts that could belong to them or to an age- and BMI-matched model and were asked to decide whether the two body parts belonged to the same or different person (same/different). For the explicit (overt) task, participants were presented with a single body part and were asked to decide whether it belonged to them or not (yes/no). Lastly, we administered self-report measures of body image and eating concerns, appearance-related worries of specific body parts and perceptual body shape distortions, to explore whether individual differences in these self-reports might affect participants’ performance at the two self-processing tasks.

In line with previous evidence showing an impairment in properly weighing sensory information in a multimodal context in later age (Marotta et al. 2018), we hypothesised that, whilst both younger and older women would display an implicit ‘self-body advantage’, with better matching of self than others’ body parts, this effect should be smaller in older women. For the explicit task, it was hypothesised that no self-body advantage, nor age effect, was expected for the explicit recognition task, reflecting no differences in the two groups in recruiting perceptual mechanisms necessary for the explicit recognition of the self.

However, both the implicit self-body advantage and the ability to explicitly recognise self-body parts should be lower in women with more body image distortions, reflecting a mismatch between the actual picture of their own body part and a distorted body representation. If this was the case, one should predict weaker self-advantage and less prompt explicit recognition for stimuli depicting body parts modified to appear thinner than the original size. This would suggest that self-body recognition is not immune to the impact of perceptual and affective components of body image.

## Methods

### Participants

Sample size calculation for both groups was based on a priori power analysis using the G*Power software (G*Power 3.1.9; Faul et al. 2007), which indicated a minimum sample of 44 participants in total as adequate for a mixed-model ANOVA design (2 groups, numerator *df* = 2) to detect, with 95% power, a moderate effect size of the variables (*f* = 0.25), setting alpha at 0.05 (two tailed) and assuming a correlation between repeated measure of 0.5. Estimate of effect size was based on the difference between young and middle-aged participants in previous study of age-related effects in body ownership (Marotta et al. 2018). A total of 53 women who self-identified as Caucasian were preselected and divided in two groups based upon age: 28 participants aged 20–37 years (*M* = 25.93, SD = 4.74) were assigned to the young women group and 25 participants aged 47–68 years (*M* = 54.36, SD = 4.54) were assigned to the older, middle-aged group. Age ranges were defined according to published literature (Marotta et al. 2018; Bellard et al. 2020). We tested only female participants, given the reported higher incidence of body image concerns and eating disorders (EDs) amongst women as compared to men (Stanford and Lemberg 2012).

Details on recruitment and inclusion criteria can be found in ESM2.

### Body image disposition measures

#### The Body Uneasiness Test

The Body Uneasiness Test (BUT) was used to measure abnormality in one’s attitudes towards body image perception on a 6-point scale (0 = never to 5 = always). The BUT is broken down into two subcomponents (BUT-A and BUT-B). The BUT-A measures 5 subcomponents: weight phobia, body image concerns, avoidance, compulsive self-monitoring and depersonalisation. Please note that for the purpose of this investigation, we focussed on the Global Severity Index (GSI, the average rating of all 34 items constituting the BUT-A), which indicates severity of elevated body image concerns and eating behaviours. Non-clinical samples have been found to score on the GSI a mean value of 0.90 (SD = 0.81), for a female population aged 40–65 years, and a mean of 1.32 (0.91), for a female population aged 18–39 years (Cuzzolaro et al. 2006). The BUT-B investigates specific worries about particular body parts, shapes or functions (e.g. mouth or skin). Please note that, whilst we reported scores of worries for each body part (see Table 1), here we considered a global measure, namely the Positive Distress Symptom Index (PDSI, the average rating of those items constituting the number of symptoms rated higher than zero) when assessing correlations with behavioural measures. Overall, the BUT has shown good internal consistency as Cronbach’s alpha coefficient is = 0.90 (Cuzzolaro et al. 2006). In this particular sample, the BUT scales had very good internal consistency (BUT-A, Cronbach’s alpha = 0.955 and BUT-B, Cronbach’s alpha = 0.940).

**Table 1:** Mean and standard deviation (in brackets) of demographics and self-report questionnaires scores for young and middle-aged women. The far-right column represents a comparison of differences between both groups for demographic information and subscales of each self-report questionnaire

	Young (*n* = 28)	Middle-aged (*n* = 25)	Young vs. middle-aged
Age	25.93 (4.74)	54.36 (4.54)	*t*_(51)_ = − 22.252, *p* < 0.001
BMI (kg/cm^2^)	24.64 (4.54)	27.71 (4.96)	*t*_(51)_ = − 2.352, *p* = 0.023
BUT-A			
Body image concern (max 5)	1.64 (0.87)	1.88 (1.03)	*t*_(51)_ = − 0.932, *p* = 0.356
Avoidance (max 5)	0.73 (0.58)	0.82 (0.70)	*t*_(51)_ = − 0.535, *p* = 0.595
Compulsive self-monitoring (max 5)	1.41 (0.93)	1.24 (0.86)	*t*_(51)_ = 0.677, *p* = 0.502
Depersonalization (max 5)	0.76 (.59)	0.67 (.75)	*t*_(51)_ = .486, *p* = 0.629
Weight phobia (max 5)	1.66 (1.03)	1.83 (1.14)	*t*_(51)_ = − .581, *p* = 0.564
Global Severity Index (max 5)	1.29 (.73)	1.37 (0.83)	*t*_(51)_ = − .404, *p* = 0.688
BUT-B			
Mouth (max 5)	1.25 (0.64)	1.42 (0.89)	*t*_(51)_ = − 0.817, *p* = 0.418
Face shape (max 5)	1.35 (0.86)	1.15 (0.81)	*t*_(51)_ = 0.863, *p* = 0.392
Thighs (max 5)	2.34 (1.06)	2.64 (1.22)	*t*_(51)_ = − 0.962, *p* = 0.340
Legs (max 5)	1.53 (0.85)	1.65 (1.35)	*t*_(51)_ = − 0.391, *p* = 0.698
Arms (max 5)	1.46 (0.98)	1.54 (1.09)	*t*_(51)_ = − 0.253, *p* = 0.801
Moustache (max 5)	0.95 (0.79)	1.07 (1.15)	*t*_(51)_ = − 0.426, *p* = 0.672
Skin (max 5)	1.66 (1.12)	1.82 (1.16)	*t*_(51)_ = 0.216, *p* = 0.830
Blushing (max 5)	1.66 (1.09)	1.48 (1.01)	*t*_(51)_ = 0.636, *p* = 0.528
Positive Symptom Distress Index (max 5)	2.02 (0.57)	2.16 (0.77)	*t*_(51)_ = − 0.774, *p* = 0.443
PFRS			
Perceived	7.11 (2.06)	8.04 (1.81)	*t*_(51)_ = − 1.740, *p* = 0.088
Actual	4.89 (1.52)	5.88 (1.70)	*t*_(51)_ = − 2.236, *p* = 0.030
Discrepancy	2.21 (1.23)	2.16 (1.41)	*t*_(51)_ = 0.150, *p* = 0.881

*BMI* body mass index, *BUT* Body Uneasiness Test, *PFRS* Photographic Figure Rating Scale

#### The Photographic Figure Rating Scale

The Photographic Figure Rating Scale (PFRS) was administered to gain information on perceptual distortions and body shape dissatisfaction in our samples of women (Gardner and Brown 2010). This scale is an improvement on the Contour Drawing Figure Rating Scale (Thompson and Gray 1995) and includes 10 images of female body shapes, from pre-established BMI categories spanning from emaciated (< 15 kg/m^2^) and underweight (15–18.5 kg/m^2^), to normal weight (18.5–24.9 kg/m^2^) and overweight (25.0–29.9 kg/m^2^), up to obese (> 30 kg/m^2^). During this task, participants were instructed to select one of the 10 test body images that they believed best represented their own shape. Based on the calculation of their BMI, researchers are able then to identify women’s actual body size and any discrepancies between women’s perceived body size and their actual body size (Swami et al. 2008). In the present study, a body size discrepancy score was then computed by subtracting perceived (actual) ratings from current ratings. Previous work has shown that the PFRS has good test–retest reliability and high construct validity (Swami et al. 2008). In this particular sample, the PFRS scale had good internal consistency (Cronbach’s alpha = 0.70).

### Stimuli preparation

**Fig. 1:**
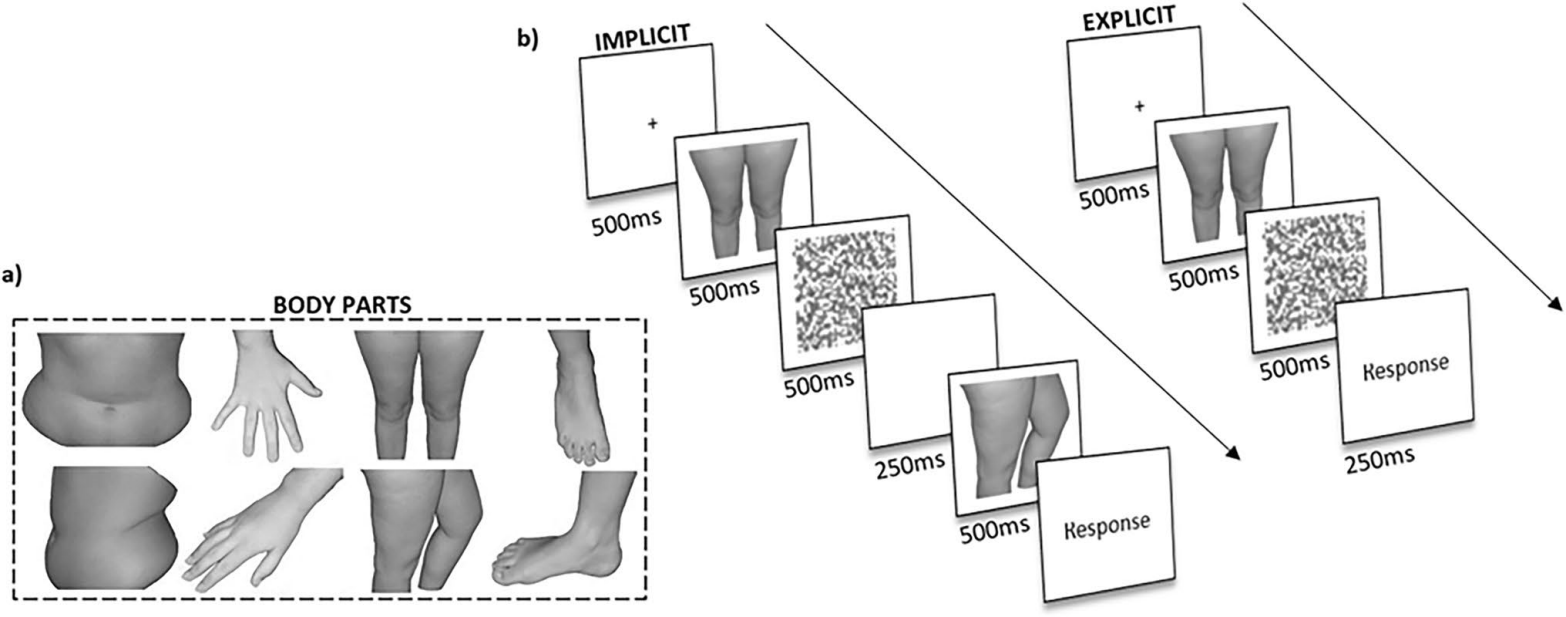
**a** Examples of body parts (stomach, hand, thighs and foot) taken for both the young and middle-aged women. Image represents both orientation of the body part, i.e. front and side view. **b** Schematic representation of the time-sequence and order of events for each trial of the implicit and explicit self-body recognition tasks

To generate the experimental stimuli, we took pictures of participants’ right hand, stomach, thighs and right foot. Each body part was photographed from both front and left-facing side views (see Fig. 1a), in a controlled (private) environment at an equal distance (approx. 2 m) from the camera lens. Whilst taking the pictures, participants were either standing against a dark background, for the stomach and thighs, or placing their hand and foot on a darkened surface. All images were taken using the same digital camera (Panasonic TZ5 Lumix) and were centred against the darkened background. This ensured all images were of the same size when presented on the screen. No flash was used to take images to ensure each image was controlled for their visual properties such as contrast and brightness. Participant’s pictures were collected on the same day as the study was taking place. In order to take images of the thighs and feet, participants were invited to wear shorts to ensure these body parts were visible. All jewellery was removed from participant when pictures were taken. No pictures of participant faces were taken, only the body parts of interest to the research. Once all pictures of the body parts were taken, these were imported and edited using Photoshop 22.0. Background was removed and replaced with a white background. All images were grey scaled to control for differences in skin tone. If any participants’ body parts contained any identifiable marking (e.g. birthmarks, moles, tattoos, scars, piercings, nail painting), these were edited out by selecting the colour of the participant’s skin and using the brush to cancel them out, to avoid participants used this as a strategy to recognise their body part. Furthermore, all pictures were altered to depict three different sizes (round, actual and slim). For the actual size, all body parts were sized 400 × 300px. For the slim size manipulation, body parts were altered to be 80% smaller in width, whilst for the rounder size manipulation, the original pictures were altered to be 120% larger. The pictures of each participant were used against three BMI-matched women of the same age group, who consented for their body part images to be used throughout the experiment. All participants’ photographs were deleted once the testing session was completed.

### General procedure

Stimulus presentation and randomisation was controlled using E-prime 2.0 software (Psychology Software Tools, Pittsburgh, PA) running on a laptop. After obtaining written informed consent, participants filled out a demographic data questionnaire followed by measurements of their height and weight. Once this step was completed, participants were shown examples of the to-be-taken body part pictures, followed by taking photographs of the specific body parts. Participants were then sat 55 cm in front of a 15.6-inch Dell monitor (resolution, 1024 × 768px; refresh frequency, 75 Hz) for the experimental tasks. Stimuli appeared in centred position of the laptop monitor on a white background.

### Implicit self-body processing task

During each trial of the implicit task, participants were first presented with a fixation cross for 500 ms. After which, a target image of either their body part or that of another woman was displayed for 500 ms on the screen. This was then followed by a visual mask, which was presented for 500 ms and followed by a 500-ms presentation of an image depicting a body part corresponding to the self or other (see Fig. 1a). Participants were then asked to report by mouse button clicking whether the two images depicted the body parts of the same person or of different persons.

### Explicit self-body processing task

For the explicit task, participants were shown a fixation cross for 500 ms. After which, they viewed a 500-ms frontal or side image of either their body part or the body part of another woman. This was then followed by a visual mask, which was presented for 500 ms (see Fig. 1b). Participants had to make a response by either pressing the left or right side of the computer mouse, to report as to whether the body part belonged to them or not (yes or no response).

For both tasks, response-button association was counterbalanced across participants. For each task, a total of 96 trials, consisting of 32 slim, 32 actual and 32 round body parts, were randomly presented. Half of the trials depicted self-body parts, whilst the remaining trials depicted the body parts of other women. Prior to this, participants were subject to 8 practice trials so that they could familiarise with the responses and timing of stimuli presentation. Task order was counterbalanced across participants. After completion of both self-processing tasks, participants filled out the BUT A/B and the PFRS scales. The experiment was completed in a single session with two short breaks, halfway during each task. Overall, testing lasted approximately 1 h.

### Data handling

Independent sample *t* test (two-tailed) was used to compare the two groups for demographic and body image variables. Accuracy and RTs for both tasks were analysed in separate three-way (2 × 3 × 2) mixed-model ANOVAs with Group (middle-aged vs. young) as the between-subjects variable and size (round vs. normal vs. slim) and identity (self vs. other) as within-subjects variables, for both the implicit and explicit tasks. All statistical analyses were performed using STATISTICA 8.0 (StatSoftInc, Tulsa, Oklahoma). All data are reported as mean (*M*) and standard error of the mean (S.E.M.). A significance threshold of *p* < 0.05 was set for all effects and effect sizes were estimated using the partial eta square measure (*ηp*^*2*^). Duncan post hoc tests were performed to follow up significant interactions. In a series of exploratory (due to the relatively small sample size for such analyses) correlation analyses, self–other difference (Δ) indexes were calculated for each task as the difference of the accuracy or RT values for self and other body parts (self–other), to explore whether any self-report questionnaires were correlated with the self-body advantage in the two tasks, using the Pearson correlation coefficient. Finally, given the well-documented increase in BMI over the lifetime (Ålgars et al. 2009) and its potential association with the outcome variables (Bellard et al. 2020), we also explored these relations as a control analysis by calculating correlations between the self–other difference (Δ) indexes and BMI in the two age groups.

## Results

### Demographic and self-report measures

Middle-aged women were significantly older and had higher BMIs and actual body silhouettes than younger women had. No age group differences were detected for all other self-report scales, pointing to comparable body image attitudes in the two groups (see Table 1).

To identify whether the two groups of women were accurate in perceiving their actual body shape, we conducted one-sample *t* tests against 0 for the body size discrepancy score of the PFRS for both groups. Both young [*t* (27) = 18.248, *p* < 0.001] and middle-aged women [*t* (24) = 22.163, *p* < 0.001] demonstrated significant overestimation of their perceived actual body size.

### Implicit self-body processing task

#### Implicit task

**Fig. 2:**
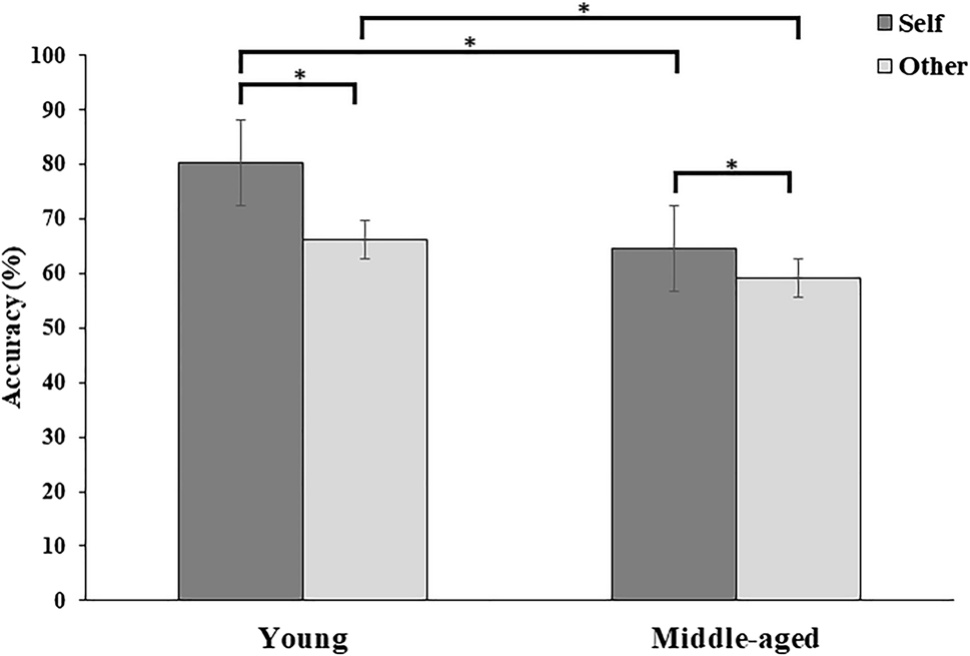
Means (standard error of the mean) for accuracy of young and middle-aged groups for the implicit self-body recognition task. Accuracy was calculated based on a percentage score to determine participants’ ability to identify if the body part belonged to the same or different person. The results are displayed as a function of body parts’ identity (self vs. other). Asterisk (*) represents a significant mean difference

The 3-way, 2 × 2 × 3 mixed ANOVA on the accuracy in the implicit self-body processing task revealed a marginally significant main effect of size [*F*_(2, 102)_ = 3.049, *p* = 0.052, *ηp*^*2*^ = 0.056], with more accurate matching of body parts when presented in their actual size (68.58 ± 1.78) compared to both their rounder (66.21 ± 1.59) or slimmer versions (65.99 ± 1.51, all *ps* < 0.033). Furthermore, significant main effects of group [*F*_(1,51)_ = 14.713, *p* < 0.001, *ηp*^*2*^ = 0.224] and identity [*F*_(1,51)_ = 53.537, *p* < 0.001, *ηp*^*2*^ = 0.512] were qualified by a significant 2-way interaction between the two factors [*F*_(1,51)_ = 8.132, *p* = 0.006, *ηp*^*2*^ = 0.138, see Fig. 2].

Post hoc comparisons revealed better performance at matching self than other body parts in both young (self: 80.43 ± 2.71% vs. other: 64.82 ± 1.79%; *p* < 0.001) and middle-aged women (self: 64.65 ± 2.87 vs. other: 57.80 ± 1.89; *p* = 0.003), supporting a consistent self-body advantage in both age groups. However, when directly comparing the amount of this advantage in the two groups, we found a greater self–other Δ for the young (Δ = 14.08, SD = 10.88) than for the middle-aged group (Δ = 5.37, SD = 8.55; *t*(51) = 3.211, *p* = 0.002). Furthermore, young women were more accurate compared to the group of middle-aged women at matching either self (young: 80.43 ± 2.71% vs. middle-aged: 64.65 ± 2.87%; *p* < 0.001) or other body parts (young: 64.82 ± 1.79%; middle-aged: 57.80 ± 1.89%; *p* = 0.05). Finally, no significant interactions were observed between group × size [*F*_(2,102)_ = 0.254, *p* = 0.776, *ηp*^*2*^ = 0.005], identity × size [*F*_(2,102)_ = 0.539, *p* = 0.585, *ηp*^*2*^ = 0.010] and group × identity × size [*F*_(2,102)_ = 0.620, *p* = 0.540, *ηp*^*2*^ = 0.012]. The lack of significance for the 3-way interaction suggests that women did not differ in their accuracy in processing their own body parts vs. the body parts of another woman depending on the body part size. This also rules out any spurious effects due to between-group differences in BMI and body silhouette.

The 2 × 2 × 3 mixed ANOVA conducted on mean *RTs* (ms) in the implicit task reported no significant main effects of size [*F*_(2,102)_ = 0.315, *p* = 0.731, *ηp*^*2*^ = 0.006], identity [*F*_(1,51)_ = 0.905, *p* = 0.346; *ηp*^*2*^ = 0.017] and group [*F*_(1,51)_ = 0.120, *p* = 0.731, *ηp*^*2*^ = 0.002]. Furthermore, no significant interactions were revealed between any of the factors of the design [group × identity: *F*_(1,51)_ = 0.012, *p* = 0.914, *ηp*^*2*^ = 0, group × size: *F*_(2,102)_ = 0.096, *p* = 0.909, *ηp*^*2*^ = 0.002, identity × size: *F*_(2,102)_ = 2.221, *p* = 0.114, *ηp*^*2*^ = 0.042 and group × identity × size: *F*_(2,102)_ = 0.192, *p* = 0.826, *ηp*^*2*^ = 0.004, see Table 2]. This suggests that group and identity modulations of accuracy could not be ascribed to speed-accuracy trade-off.

**Table 2:** Mean (standard error of the mean in brackets) of accuracy (%) and reaction times (ms) for the implicit task for the two groups of young and middle-aged women. Scores depict both the accuracy and reaction times for self vs other body parts, according to the three levels of size distortion (round, actual and slim)

	Young (*n* = 28)		Middle-aged (*n* = 25)	
	Self	Other	Self	Other
Accuracy scores				
Round	78.61 (15.86)	64.68 (9.27)	63.84 (15.66)	57.72 (14.67)
Actual	83.64 (13.77)	65.86 (11.80)	65.60 (19.45)	59.20 (13.04)
Slim	79.04 (12.87)	63.93 (8.31)	64.52 (16.09)	56.48 (12.10)
Reaction times				
Round	976.30 (43.97)	968.08 (54.01)	1006.94 (46.53)	994.27 (57.16)
Actual	965.66 (48.30)	1000.22 (45.81)	980.33 (51.12)	1027.71 (48.48)
Slim	977.38 (44.74)	995.79 (48.82)	1004.21(47.35)	1005.07 (51.66)

**Fig. 3:**
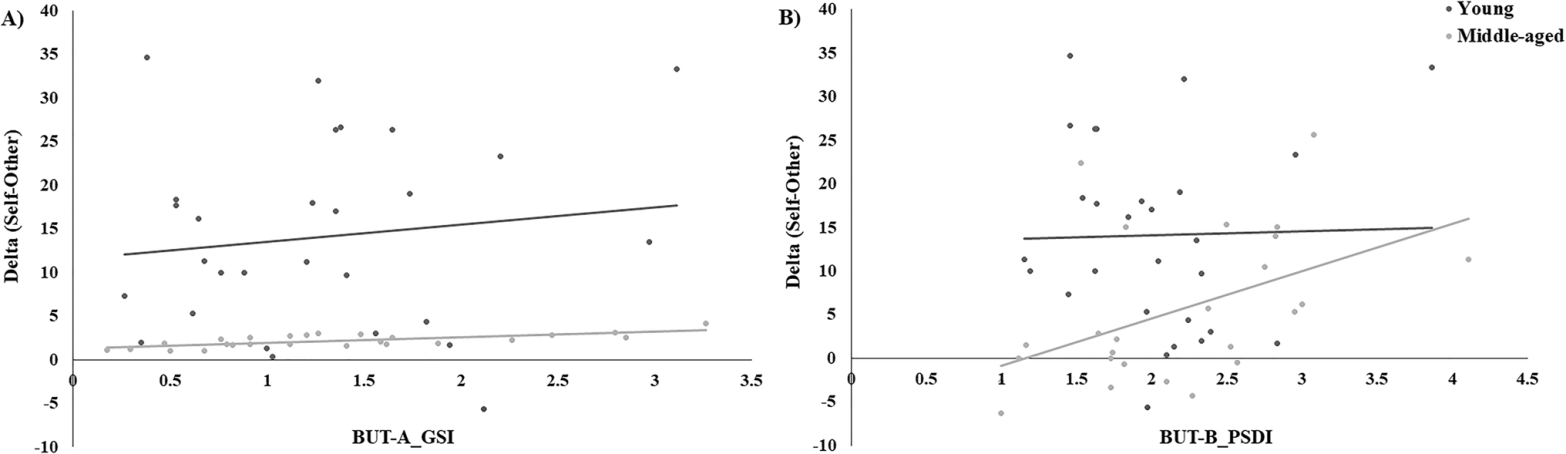
**A** Combined scatterplot for the correlation between accuracy Δ index (self–other) and the scores obtained at the Global Severity index (GSI) subscale of the Body Uneasiness Test-B (BUT-A_GSI). **B** Combined scatterplot for the correlation between accuracy Δ index (self–other) and the scores obtained at the Positive Symptom Distress Index (PSDI) subscale of the Body Uneasiness Test-B (BUT-B_PSDI). Lines represent trendline. Dark grey circles represent the young women group and dark grey circles represent the middle-aged women group

Because of the small sample size, we conducted exploratory Pearson’s correlation analyses which demonstrated that the accuracy Δ index of middle-aged women showed a significant positive correlation with both their BUT-A GSI (*r* = 0.462, *p* = 0.020) and their BUT-B PSDI (*r* = 0.486, *p* = 0.014) (see Fig. 3). On the contrary, no correlations were observed between the accuracy Δ index and older women’s BMI (*r* = 0.294, *p* = 0.154), nor with the discrepancy score obtained at the PFRS scale (*r* = 0.012, *p* = 0.954). For the young group, no significant correlations were observed between the individual accuracy Δ index and their BMI or any of the scores obtained at the self-reports (all *ps* > 0.501). No significant correlations were obtained for the RTs Δ index of either group (all *ps* > 0.353).

### Explicit self-body processing task

The 2 × 2 × 3 mixed ANOVA for the accuracy (%) in the explicit self-body processing task revealed a main effect of group [*F*(1, 51) = 15.463, *p* < 0.001, *ηp*^2^ = 0.233], with younger women being overall more accurate than older women in recognising body parts (young: 81.96 ± 3.09%; middle-aged: 65.16 ± 3.20%). A significant main effect of size was also observed [*F*(2, 104) = 6.145, *p* = 0.003, *ηp*^2^ = 0.108]. Post hoc comparisons revealed that women were significantly less accurate in recognising slim-distorted body parts (71.08 ± 2.22%), compared to round-distorted (74.40 ± 2.24%) and actual body parts (74.02 ± 2.47%, all *p*s < 0.002). No significant difference was observed between round and actual body parts (*p* = 0.683). No significant main effect was revealed for identity [*F*(1,51) = 0.605, *p* = 0.440, *ηp*^2^ = 0.012]. Furthermore, the interaction between identity × size was only marginally significant [*F*(2,102) = 3.796, *p* = 0.057, *ηp*^2^ = 0.068]. This was explained by a significant lower accuracy in recognising slim distorted body parts when belonging to the self compared to all other conditions of self and other size-distorted body parts (all *p*s < 0.019). No significant interactions of group × size [*F*(2,102) = 1.911, *p* = 0.153, *ηp*^2^ = 0.036], group × identity [*F*(1,51) = 3.200, *p* = 0.080, *ηp*^2^ = 0.059] or group × identity × size [*F*(2, 102) = 0.217, *p* = 0.805, *ηp*^2^ = 0.004] were reported. The lack of significant interactions with group suggests that the weakness in explicitly recognising thinner self-body parts as compared to either rounder or actual-size body parts was comparable in the two age groups. This again rules out any spurious effects due to between-group differences in BMI and body silhouette.

The 2 × 2 × 3 mixed ANOVA conducted on mean RTs (see Table 3) in the explicit self-processing task revealed a significant main effect of Identity [*F*(1,49) = 5.208, *p* = 0.027; *ηp*^2^ = 0.096], which was corroborated by a significant 2-way interaction of identity × group [*F*(1,49) = 5.297, *p* = 0.026; *ηp*^2^ = 0.098]. Post hoc comparisons revealed that middle-aged women were significantly faster in recognising self (866.30 ± 59.93 ms) than other women’s body parts (942.31 ± 58.18 ms, *p* = 0.002), whilst no such difference was noted for the younger women (self: 1009.45 ± 54.32 ms vs. 1009.13 ± 52.73 ms, *p* = 0.989). Furthermore, younger women did not differ from middle-aged women in processing either self (young: 1009.45 ± 54.32 vs. middle-aged: 866.30 ± 59.93, *p* = 0.106) or other (young: 1009.13 ± 52.73 vs. middle-aged: 942.31 ± 58.18, *p* = 0.406) body parts. Taken together, these effects point to an age-related modulation of the speed-accuracy trade for self-recognition, since middle-aged women were less accurate but responding much faster than young women in identifying body parts belonging to the self, suggesting ageing may change the strategic processing of self-body images. No other significant main effects or interactions were revealed between any of the factors of the design [age: *F*(1,49) = 1.813, *p* = 0.184, *ηp*^2^ = 0.036, size: *F*(2,98) = 0.309, *p* = 0.735, *ηp*^2^ = 0.006, group × size: *F*(2,98) = 0.061, *p* = 0.941, *ηp*^2^ = 0.001, identity × size: *F*(2,98) = 0.979, *p* = 0.379, *ηp*^2^ = 0.020 and group × identity × size: *F*(2,98) = 0.512, *p* = 0.601, *ηp*^2^ = 0.010, see Table 3].

**Table 3:** Mean (standard error of the mean in brackets) of accuracy (%) and reaction times (ms) for the explicit task for the two groups of young and middle-aged women. Scores depict both the accuracy and reaction times for self vs other body parts, according to the three levels of size distortion (round, actual and slim)

	Young (*n* = 28)		Middle-aged (*n* = 23)	
	Self	Other	Self	Other
Accuracy scores				
Round	79.11 (19.54)	89.11 (15.91)	69.04 (20.49)	66.91 (20.10)
Actual	80.50 (18.30)	85.50 (17.31)	69.78 (24.82)	66.91 (19.62)
Slim	72.07 (23.50)	85.50 (16.91)	63.04 (23.38)	69.83 (23.01)
Reaction times				
Round	1010.46 (54.23)	1010.66 (54.50)	866.19 (59.84)	943.90 (60.14)
Actual	1004.80 (57.21)	1007.91 (53.18)	848.73 (63.12)	947.01 (58.67)
Slim	1013.11 (55.35)	1008.82 (53.63)	883.97 (61.07)	936.01 (59.18)

Because of the small sample size, we conducted exploratory Pearson’s correlation analyses which indicated a significant negative association between the discrepancy scores obtained at the PFRS and the mean accuracy for slim distorted body parts belonging to the self in the middle-aged (*r* =  − 0.562, *p* = 0.003), but not in the younger women (*r* =  − 0.080, *p* = 0.685) group. On the contrary, neither the accuracy nor the RTs Δ indices correlated with BMI or any of the self-reported measures in any of the two groups of women (all *r*s <  − 0.331, all *p*s > 0.107).

## Discussion

In keeping with previous findings that individuals are more accurate in implicitly identifying body parts belonging to the self, compared to that of another person (Frassinetti et al. 2008, 2009, 2011), we found that both groups of women were more accurate in implicitly processing self than other body parts, thus demonstrating the so-called self-body advantage. This is not surprising given the constant exposure to our own body, which makes it much easier and quicker to identify our body amongst the body parts of other women. Indeed, a mental representation of the body and its parts is recalled from memory, which is constantly updated when changes to the body occur (Ionta et al. 2012).

Nevertheless, young women had a greater ‘self-body advantage’ in the implicit task compared to middle-aged women. To the best of our knowledge, this is the first study of this kind showing that, although the self-body advantage seems to be preserved across lifespan, older women might show a weakening in implicit self-body representation. A possible explanation for this effect is that, whilst it is known that implicit self-body recognition relies upon a rich array of information coming from interoception, vision, somatosensory, proprioceptive and motor information (Myers and Sowden 2008), the ability of efficiently integrating such information to form a coherent sense of self might naturally decline with age (Marotta et al. 2018). In particular, the implicit model of the metric proprieties of the body (i.e. information about the size and the shape of different body parts), which is stored in the brain and is updated through online peripheral signals (Longo and Haggard 2010, 2012; de Vignemont 2010; Longo et al. 2010; Medina and Coslett 2010; Serino and Haggard 2010), might be subject to greater distortion with ageing. Furthermore, a decline in the processing of sensorimotor information, which is required to maintain an updated body representation, has been already described in older adults (Costello and Bloesch 2017; Kuehn et al. 2018). Indeed, the ability to operate mental imagery of body parts, which requires the creation, maintenance and activation of an internal representation of the body (Kaltner et al. 2014), has been reported to decline with ageing as compared to mental imagery of other objects (Devlin and Wilson 2010; De Simone et al. 2013). Accordingly, the smaller self-body advantage in older compared to younger women might reflect a distortion in their implicit body metric representation.

Interestingly, we also found that the advantage in recognising self vs. other body parts was positively associated to the severity of body image concerns and eating behaviours (BUT-GSI) and to the intensity of worries for specific body parts (BUT-PSDI), specifically in older but not in younger women. Therefore, older women displaying more elevated body image concerns and distress for the appearance and function of specific body parts also showed a greater advantage when implicitly recognising self vs. other body parts. However, whilst these results are interesting and pave the way for further systematic investigations of the moderating role of negative body image in self-body recognition, our findings should be interpreted with caution given their exploratory nature and the relatively small sample size for such analyses, which in turn might prevent achieving stable estimates for correlations (Schönbrodt and Perugini 2013) and therefore generalisability of our results to a broader population. Regardless, it is important to note that our samples of young and middle-aged women displayed comparable levels of body image concerns (BUT-GSI) and worries for specific body parts (BUT-PSDI), thus ruling out that between-group differences in self-body processing were due to age-related changes of body image dispositions. Yet, although we showed that varying levels in adiposity, as indexed by BMI, were not linearly associated with outcome measures of self-body recognition in the two age groups, we cannot rule out that participants’ BMI might have affected their responses if direct and indirect pathways were to be systematically investigated by means of moderation/mediation analyses. As also suggested by a previous study by our group (see Bellard et al. 2020), which reported that distortions in the perceptual components of middle-aged women’s body image are best explained by a combination of BMI, body part concerns and age group, in future BMI should be considered to fully appreciate the intricate patterns of age, BMI and body image negative disposition across lifespan.

Previous investigations have reported contrasting results regarding the importance that middle-aged women place on their bodily appearance. On one hand, some studies demonstrated a decreased appearance investment (Kilpela et al. 2015; Tiggemann 2004) and a shift towards the appreciation of functionality of the body (i.e. what body is capable of doing—rather than how it looks, Reboussin et al. 2000; Alleva et al. 2015, 2016); on the other hand, other studies suggest that older women can experience negative feelings and attitudes towards their body, such as body dissatisfaction and drive for thinness (Bane and McAuley 1998; Longo et al. 2009) along with over-estimation of their body size (Bellard et al. 2020), similarly to what happens in younger women. This level of dissatisfaction with the body remains constant, but it is the level of the importance placed on the body which decreases as women get older (Tiggemann and Lynch 2001). Our results of comparable body image concerns and worries for specific body parts in our samples of young and middle-aged women seem to speak in favour of the latter idea with young and older women placing comparable affective importance in the appearance of specific body parts. Furthermore, our finding of a positive correlation between the implicit self-body advantage and eating and body image concerns suggests that whilst ageing may hinder an implicit access to body representation, body image concerns and eating problems may increase attention to bodily changes and boost the accuracy in processing self-body parts when placed amongst other women.

This finding contrasts with recent study by Campione et al. (2017), which compared women with disordered eating and age- and gender-matched controls in their ability to implicitly recognise their own body. The results showed that women with EDs differed from controls in their ability to process self-stimuli vs. non-self-stimuli and did not demonstrate the classic self-body advantage. In a similar vein, a previous study by Urgesi et al. (2011) reported that, during a laterality judgement task, women suffering from bulimia nervosa showed an impairment in the ability to simulate a motor mental rotation of their own body in order to assume the perspective of the displayed body figure. Taken together, these results support the idea of an altered body schema representation in EDs, which might lead to weaker implicit self-body processing.

Whilst these findings of altered body schema in ED patients would suggest that higher body image concerns predict lower self-body advantage, it is noteworthy that our implicit self-body processing task required the visual discrimination of single body parts, a task in which women with EDs have been shown to outperform age-matched controls (Urgesi et al. 2012). In this regard, although prior to uploading images all identifiable marks (e.g. tattoos, scars, moles) were edited out using Photoshop to prevent participants using these cues to identify their body parts, it is difficult to be sure that all identifiable features were excluded from the image. Participants, thus, and particularly those middle-aged women with greater body image concerns, may have looked at a very small feature which makes their body parts identifiable compared to other women. Accordingly, a bias towards the local processing of body parts’ details and deficits in configural processing of the global body shape has been reported in individuals with an ED, and in particular in those with Anorexia Nervosa (Urgesi et al. 2014). Thus, it is possible that the relation between body image concerns and the self-body advantage in middle-aged, but not younger, women might reflect a greater relying on distinctive marks which makes them different to identify their own body parts. This is also in keeping with the age-related changes in the accuracy and speed of processing body stimuli during the explicit self-body processing task. Indeed, as compared to younger women, middle-aged women were overall less accurate and quicker in determining whether a body part belonged to them or not. Furthermore, they were also particularly quicker than younger women in reporting that a body part belonged to them rather than to another woman. The trade-off between accuracy and speed of responses for this age-related effect does not support a general reduction of self-body explicit recognition with ageing, but it may point to a change in the perceptual strategy used to identify the self, likely favouring the detection of single features rather than the processing of the global figure.

Despite the fact that the two groups differed in their overall perceptual processing of a body parts to detect whether it belonged to them or not, both groups were less accurate in explicitly recognising self-body parts that were manipulated to appear thinner as compared to their actual size. This difficulty was instead not evident when body parts were manipulated to appear rounder than their actual size, since no difference between the processing of rounder and actual sized body parts was detected. This effect might occur due to a mismatch between the actual size of the physical body and an overestimated size of the stored self-body representation. Accordingly, we found, at least amongst middle-aged women, a negative correlation between the ability to recognise self-body parts and the PFRS discrepancy scores, which measures perceptual distortions of the actual whole body. Indeed, those middle-aged women who reported a greater perceptual discrepancy between the current and the perceived whole-body shape (with greater overestimation) were also less able to discriminate self from others’ slim body parts.

## Limitations

Certain limitations of the present study should be acknowledged. First of all, although we recruited women with no current and history of any psychiatric disorders (including EDs), we did not conduct a structured interview to systematically exclude diagnosed cases of EDs. At present, we cannot, thus, rule out that age-related effects on self-body processing might be ascribed to more complex, profound distortions of the self that characterise EDs, such as anorexia nervosa, which might have been hidden in our sample of healthy women. Second, the type of images used in the current study were all grey scaled. Although this prevented skin tone being a factor, which helped women identify their own body parts, these images are less life-like and are not a full depiction of the mental representation an individual would have about their body parts. As suggested by Frassinetti et al. (2011), these images may have made the task slightly more difficult for participants to identify the self. Therefore, future studies could look into using coloured versions compared to grey-scaled images to see whether changes in colour enhance task difficulty and affect overall self-body recognition accuracy. In addition, it may also be beneficial to counterbalance both the computerised tasks and the questionnaire administration, so as to avoid any potential bias in responses; i.e. participants guess the true aims of the experiment if the task is always completed first (Gove and Geerken 1977; McCambridge et al. 2012). In the future, it will also be beneficial to investigate whether similar findings are observed in women of different ethnicities to identify whether the ‘self-body advantage’ is more prevalent in younger women compared to middle-aged women in ethnicities other than Caucasian. Also, we did not control for other demographic variables (e.g. relationship status, education) that may co-vary with age of the two groups, thus preventing us from excluding that between-group differences could be influenced by factors other than age. Finally, this investigation did not explore the role of self-esteem in self-body recognition. A study from Richetin et al. (2012) has reported that implicit and explicit self-esteem provide important but different contributions to self-body recognition abilities, thus suggesting not only that individual differences play a role in cognitive functions, but also that self-body recognition could constitute an additional cognitive indicator in the assessment of body image. Therefore, it would be useful for future investigations to explore how levels of self-esteem impact upon self-body recognition, particularly given the fact the middle-aged compared to younger women are more prone to lower self-esteem due to significant bodily changes as a result of ageing (Bosworth et al. 2001; Dennerstein et al. 2000; Elavsky 2010).

## Conclusions

To conclude, our results provide, for the first time, evidence that age is a factor contributing towards alterations of the implicit and explicit perceptual processing of body parts. Furthermore, although preliminary, our findings hint at an association between perceptual and cognitive distortions of body image and the perceptually based mechanisms required in self-body recognition across the lifespan. This suggests that ageing may not only affect the affective and cognitive components of (negative) body image, but also alter the perceptual processing of body dimensions, which might find its source in a multisensory network underpinning the bodily self and self-awareness. Overall, our research might help to emphasise to both health care professionals and the public that body image concerns are a problem not only for younger women, but they are also present in women of middle age. Furthermore, disordered eating prevention programmes, or therapeutic approaches for several mental disorders for which negative body image is a focus (including eating disorders and body dysmorphic disorder), could benefit from taking into account a more comprehensive perspective of the factors that might contribute to alterations of the ability to recognise the ageing body and the relative negative impact of perceptual and affective components of body image in younger and older populations.

## Supplementary Information

Below is the link to the electronic supplementary material.

Supplementary file1 (XLSX 30 KB)

Supplementary file2 (DOCX 15 KB)

## Data Availability

The datasets analysed during the current study are available in the form of supplementary materials.
