# Supplementary material for: Self-body recognition and attitudes towards body image in younger and older women
Source: Arch Womens Ment Health. 2021 Jul 31;25(1):107–19. doi: 10.1007/s00737-021-01164-x (PMC8784361; doi:10.1007/s00737-021-01164-x)
Supplement: Supplementary file 2 — Supplementary file2 (DOCX 15 KB) [file 737_2021_1164_MOESM2_ESM.docx]

**Details on recruitment and inclusion/exclusion criteria**

Participants were recruited internally among members of *** staff and students through word of mouth and externally via a poster advertisement and social media. Younger women were also recruited internally through the *** Psychology SONA participation scheme for undergraduate Psychology students. Middle-aged women that had been in prior lab studies were also contacted from our database of previous study participants (Psychology Research Participants Panel). All women (self)reported not to have any history of EDs and did not suffer from any neurological or psychiatric disorders. Furthermore, they had no vision problems that could not be corrected (glasses and contact lenses were acceptable) and were not pregnant. Each woman’s actual BMI was physically measured and calculated from their weight and height by means of a calibrated bioimpedance digital scale (OMRON BF511) and a stadiometer for height. To make sure that our samples of women were BMI-matched with respect to the other models, younger and older participants were eligible only if their BMIs was between 17 and 28. All women were right-handed apart from 5 Young and 3 Middle-aged. All women provided written and verbal informed consent prior to taking part to this experiment. Participants received written and oral information about the study, clarifying the aim to compare self-body perception in middle-aged and young women and that to do so, pictures of their body parts should be taken and used in a behavioural computerized task, followed by the administration of a series of questionnaires about eating attitudes and cultural standards of body attractiveness. As an incentive, participants either received SONA (participation point scheme) points (if undergraduate students) or £10 in shopping vouchers. The study experimental procedures and methods were fully approved by *** Research Ethics Board and complied with the ethical standards of the 1964 Declaration of Helsinki.
